# Establishing best practices in photoplethysmography signal acquisition and processing

**DOI:** 10.1088/1361-6579/ac6cc4

**Published:** 2022-05-25

**Authors:** Peter H Charlton, Kristjan Pilt, Panicos A Kyriacou

**Affiliations:** 1 Department of Public Health and Primary Care, University of Cambridge, United Kingdom; 2 Research Centre for Biomedical Engineering, City, University of London, United Kingdom; 3 Department of Health Technologies, Tallinn University of Technology, Ehitajate tee 5, 19086 Tallinn, Estonia

## Abstract

Photoplethysmography is now widely utilised by clinical devices such as pulse oximeters, and wearable devices such as smartwatches. It holds great promise for health monitoring in daily life. This editorial considers whether it would be possible and beneficial to establish best practices for photoplethysmography signal acquisition and processing. It reports progress made towards this, balanced with the challenges of working with a diverse range of photoplethysmography device designs and intended applications, each of which could benefit from different approaches to signal acquisition and processing. It concludes that there are several potential benefits to establishing best practices. However, it is not yet clear whether it is possible to establish best practices which hold across the range of photoplethysmography device designs and applications.

This Editorial considers whether it would be possible and beneficial to establish best practices for acquiring and processing photoplethysmography signals.

Photoplethysmography is an optical technique which provides non-invasive measurements of the arterial pulse wave, which is related to the blood volume change in the observed microvascular tissue. The photoplethysmogram (PPG) signal is already widely utilised by clinical devices such as pulse oximeters (Alian and Shelley), and wearable devices such as smartwatches (Charlton and Marozas). Photoplethysmography holds great promise for health monitoring in daily life. Indeed, several potential applications of photoplethysmography were presented in 2021 alone in *Physiological Measurement*, including: blood pressure monitoring (Esmaelpoor *et al* 2021, Xing *et al* 2021); detecting peripheral arterial disease (Allen *et al* 2021); sleep staging (Li *et al* 2021); screening for sleep apnea and cardiovascular disease (Behar *et al* 2020, Ouyang *et al* 2021); and detecting driver sleepiness (Hultman *et al* 2021).

Despite the widespread use of photoplethysmography, best practices have not yet been established for acquiring and processing photoplethysmography signals. This may in part be due to the diversity of photoplethysmography device designs, ranging from smartwatches to earbuds, and applications, ranging from oxygen saturation measurement in clinical practice to heart rate monitoring during exercise (Charlton *et al* 2022). Potentially, the best approach to signal acquisition and signal processing could differ between each device design and application. Nonetheless, there could be benefits to establishing best practices, such as establishing hardware configurations that consistently provide high quality signals, and establishing signal processing algorithms that can accurately derive parameters from a variety of PPG signals. This is illustrated by the findings of Liu *et al* in their recent article in *Physiological Measurement*. They found that the use of different PPG signal filtering settings can result in different measurements being obtained from PPG pulse wave analysis. Based on this, they highlighted the potential benefits of the ‘standardisation’ of PPG filtering (Liu *et al* 2021). In this case, establishing best practices for filtering PPG signals would have the benefit of allowing pulse wave indices to be compared between studies and between devices. However, this may not be straightforward as different filtering settings may be required for different applications, such as heart rate monitoring (which uses the fundamental frequency of the PPG, ≈0.5–3Hz) and blood pressure assessment (which uses higher frequency content).

Potential areas in which best practices could be established include factors relating to device design (hardware and software) and measurement protocols (recording setting and duration). These are summarised in figure 1, and now described.

**Figure 1.**
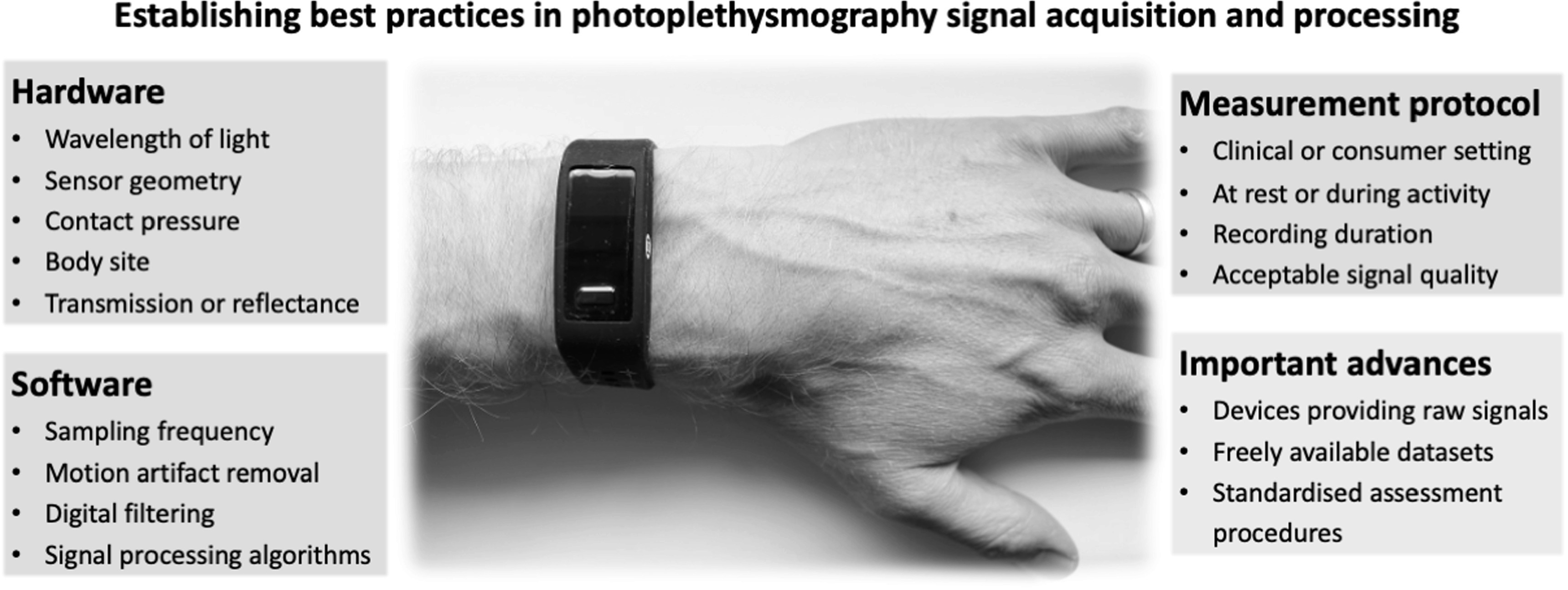
Factors influencing photoplethysmography measurements, and important advances towards establishing best practices. Source: This Max Health Band image has been obtained by the authors from the Wikimedia website where it was made available by Peter H Charlton under a CC BY 4.0 licence. It is included within this article on that basis. It is attributed to Peter H Charlton.

Several factors in the hardware design influence the PPG signal (Charlton and Marozas, Lemay *et al*), and are therefore potential areas in which best practices could be established. Firstly, the wavelength of emitted light determines the depth of light penetration, and consequently the level of the vasculature contributing to the PPG signal (Liu *et al* 2019), which influences signal quality (Fallow *et al* 2013). Current best practice is to use longer wavelengths (e.g. infrared) for transmission photoplethysmography as these penetrate deeper (Anderson and Parrish 1981), and shorter wavelengths (e.g. green) for reflectance photoplethysmography as these produce higher signal quality for heart rate measurement (Matsumura *et al* 2020). However, this practice may need to be revisited as the use of green light has been found to result in less accurate heart rate monitoring in subjects with darker skin tones (Fine *et al*). Secondly, in reflectance photoplethysmography the signal quality is influenced by the geometry of the light emitter, light detector, and sensor casing. Current best practice is to design the surrounding casing to eliminate ambient light as far as possible (Abay and Kyriacou). In the future this may be extended to using geometries in which the LED surrounds the photo detector, as these have been found to give higher signal quality (Khan *et al* 2019). Thirdly, the contact pressure applied by the device to the skin impacts the shape of the PPG pulse wave (Chandrasekhar *et al* 2020) and consequently its second derivative (Grabovskis *et al* 2013). Best practice in the area of contact pressure has not yet been established: higher pressures may reduce probe-tissue movement artifact, and have been found to increase the accuracy of PPG-based heart rate monitoring (Scardulla *et al* 2020). However, it is not clear whether such pressures would be suitable for long-term monitoring. Ideally, the contact pressure should remain constant when analysing pulse wave shape, such as when tracking changes nocturnal changes in blood pressure (Radha *et al* 2019). Fourthly, the body site chosen for PPG measurement influences pulse wave shape (Hartmann *et al* 2019), and the utility of the acquired signal (Charlton and Marozas). Best practice has not yet been established in this area: in clinical devices the finger is often used (Alty *et al* 2007), whereas in consumer devices the wrist is often used due to user preference (Prinable *et al* 2017). In summary, the challenge of establishing best practices is not trivial, as several factors can influence the PPG signal, and it is likely that different device configurations would be best suited to different applications.

The software used in PPG devices influences the PPG signal and the parameters derived from it, and therefore presents potential areas in which to establishing best practices. Firstly, there is a compromise between increasing the sampling frequency to capture details of the shape of PPG pulse waves, and reducing it to reduce power consumption (Lee *et al* 2018). Best practices differ between applications, with minimum acceptable sampling frequencies of 10, 16, and 25 Hz reported for heart rate, respiratory rate, and pulse rate variability measurements respectively (Wolling and Van Laerhoven, Charlton *et al* 2017, Choi and Shin 2017). Secondly, different approaches can be used to remove motion artifact, ranging from eliminating periods of motion (Guo *et al* 2021), to denoising the PPG (Zhang *et al* 2015), to cancelling motion artifact using a reference accelerometer or gyroscope signal (Marozas and Charlton 2021). Here, best practices also differ between applications: in hospital monitoring it has been proposed that periods of motion should be eliminated from analyses (Orphanidou *et al* 2015), whereas in exercise monitoring the alternative approaches of denoising the PPG or cancelling motion artifact are used (Zhang *et al* 2015). Whilst it may be challenging to develop a universal strategy to PPG signal quality assessment, recent work has demonstrated that a single approach can perform well across different heart rhythms and different PPG devices (Mohagheghian *et al*). Thirdly, the analog and digital filtering used to pre-process signals influences both the amplitudes and timings of PPG pulse wave features (Liang *et al* 2018, Liu *et al* 2021). For instance, an optimal low-pass filter cut-off of 6 Hz has been proposed to preserve the higher harmonic components of the PPG, and minimise variability in indices calculated from its second derivative (Pilt *et al* 2013). Fourthly, the choice of signal processing algorithm used to estimate a physiological parameter from the signal can greatly influence the accuracy and precision of the parameter (Charlton *et al* 2016). Best practices for deriving pulse wave features from finger PPG signals have been proposed (Elgendi 2014, Elgendi *et al* 2014). However, best practices have not yet been established for signals acquired at the wrist, which differ from finger signals (Rajala *et al* 2018). Similarly, it could be beneficial to optimise neural network architectures for PPG analyses, building on existing architectures (Li *et al* 2021). Further work is also required to identify the best pulse wave features for different tasks from amongst the wide range of features proposed in the literature (Charlton *et al* 2018, Lin *et al* 2020). For instance, recent studies have investigated the best features for blood pressure estimation (Xing *et al* 2021) and pulse rate variability analysis (Peralta *et al* 2019). The best algorithm design may also depend on a subject’s characteristics, as shown by recent proposals of different blood pressure estimation algorithms for subjects of different ages (Xing *et al* 2020) and subjects of different blood pressure categories (Khalid *et al* 2020). In summary, it may be difficult to establish best practices for the software used in PPG devices, as the best approach may vary according to the sensor configuration, application, and subjects being monitored.

A further area in which best practices could be established is the protocols used to obtain PPG measurements, where best practices could be used to obtain repeatable and reproducible measurements. Measurement protocols can be tightly controlled in clinical settings, where consideration can be given to room temperature, subject position, and the duration of rest prior to measurement (Allen and Hedley 2019). However, protocols cannot be so tightly controlled when obtaining measurements from consumer devices in daily life. Nevertheless, measurements can be obtained in a repeatable manner during periods of rest, such as resting and night-time resting heart rates (Mishra *et al* 2020, Radin *et al* 2020). Future work may consider the required recording durations and acceptable levels of signal quality to estimate different physiological parameters from the PPG (Huthart *et al* 2020). Whilst it is possible to obtain some parameters during exercise (e.g. heart rate) (Zhang *et al* 2015), it may only be possible to obtain other parameters accurately whilst at rest (e.g. those derived from the second derivative of the PPG, such as the aging index) (Takazawa *et al* 1998).

It is clear that there are several potential areas in which best practices could be established for the acquisition and processing of PPG signals. However, it is not yet clear whether it would be possible and beneficial to establish best practices. On the one hand: it may not be possible to establish best practices as they may vary greatly between device designs and applications; it may not be possible to use them widely if they are patented; and, they may not be beneficial if they don’t substantially improve device performance. On the other hand, establishing best practices could: reduce the time taken to design and manufacture devices; ensure PPG-based measurements are as accurate and reproducible as possible; and, help advance the field as researchers and developers could build on existing best practices when making novel developments.

Several advances could aid research into determining whether it would be possible and beneficial to establish best practices for PPG signal acquisition and processing. Firstly, wearable devices which provide the raw PPG signal are invaluable for such research, as demonstrated through the use of the Empatica E4 wristband in many research studies (McCarthy *et al*). Whilst several research devices can provide the raw PPG signal (Charlton *et al* 2022), large-scale studies could be conducted more easily in daily life if consumer devices were similarly able to provide raw PPG signals. Secondly, freely available datasets allow researchers to benchmark their own PPG signal processing algorithms against others on a common dataset. Several such datasets are available (Charlton *et al* 2022), including: the WeSAD and PPG-DaLiA datasets, acquired using an Empatica E4 device in healthy subjects (Schmidt *et al*, Reiss *et al* 2019); and the VitalDB and the MIMIC Waveform databases, acquired from critically-ill patients (Johnson *et al* 2016, Lee and Jung 2018). However, there are limitations to current datasets: they are often collected from either healthy volunteers or a particular patient population, rather than a broad cross-section of society; they often contain PPG signals acquired by only one device, rather than signals acquired using different hardware configurations; and they are often recorded in either laboratory or clinical settings, but few are recorded in daily life. Thirdly, there is a need for widely accepted validation protocols with which to assess the performance of PPG-based devices. Such protocols already exist for devices measuring blood pressure and heart rate (Stergiou *et al* 2018, Mühlen *et al* 2021). However, different standards may be required for different applications, such as varying the accuracy and data availability thresholds according to the intended use case and measurement scenario (Consumer Technology Association 2018, Mukkamala *et al* 2021).

To conclude, there are several potential benefits to establishing best practices for acquiring and processing PPG signals. However, it is not yet clear whether it is possible to establish best practices which hold across the range of PPG device designs and applications. Therefore, much further work is required to investigate whether it would be possible and beneficial to establish best practices, and to understand how they may differ between device designs and intended applications.

## References

[pmeaac6cc4bib18] Abay T Y, Kyriacou P A, Kyriacou P A, Allen J (2022). Photoplethysmography in oxygenation and blood volume measurements. Photoplethysmography: Technology, Signal Analysis, and Applications.

[pmeaac6cc4bib1] Alian A A, Shelley K H, Kyriacou P A, Allen J (2022). PPG in clinical monitoring. Photoplethysmography: Technology, Signal Analysis and Applications.

[pmeaac6cc4bib47] Allen J, Hedley S (2019). Simple photoplethysmography pulse encoding technique for communicating the detection of peripheral arterial disease - a proof of concept study. Physiol. Meas..

[pmeaac6cc4bib5] Allen J, Liu H, Iqbal S, Zheng D, Stansby G (2021). Deep learning-based photoplethysmography classification for peripheral arterial disease detection: a proof-of-concept study. Physiol. Meas..

[pmeaac6cc4bib29] Alty S R, Angarita-Jaimes N, Millasseau S C, Chowienczyk P J (2007). Predicting arterial stiffness from the digital volume pulse waveform. IEEE Trans. Biomed. Eng..

[pmeaac6cc4bib15] Anderson R R, Parrish J A (1981). The optics of human skin. J. Invest Dermatol.

[pmeaac6cc4bib8] Behar J A (2020). Remote health diagnosis and monitoring in the time of COVID-19. Physiol. Meas..

[pmeaac6cc4bib20] Chandrasekhar A, Yavarimanesh M, Natarajan K, Hahn J-O, Mukkamala R (2020). PPG sensor contact pressure should be taken into account for cuff-less blood pressure measurement. IEEE Trans. Biomed. Eng..

[pmeaac6cc4bib26] Charlton P H, Bonnici T, Tarassenko L, Alastruey J, Clifton D A, Beale R, Watkinson P J (2017). Extraction of respiratory signals from the electrocardiogram and photoplethysmogram: technical and physiological determinants. Physiol. Meas..

[pmeaac6cc4bib36] Charlton P H, Bonnici T, Tarassenko L, Clifton D A, Beale R, Watkinson P J (2016). An assessment of algorithms to estimate respiratory rate from the electrocardiogram and photoplethysmogram. Physiol. Meas..

[pmeaac6cc4bib41] Charlton P H, Celka P, Farukh B, Chowienczyk P, Alastruey J (2018). Assessing mental stress from the photoplethysmogram: a numerical study. Physiol. Meas..

[pmeaac6cc4bib10] Charlton P H, Kyriacou P A, Mant J, Marozas V, Chowienczyk P, Alastruey J (2022). Wearable photoplethysmography for cardiovascular monitoring. Proc. IEEE.

[pmeaac6cc4bib2] Charlton P H, Marozas V, Kyriacou P A, Allen J (2022). Wearable photoplethysmography devices. Photoplethysmography: Technology, Signal Analysis and Applications.

[pmeaac6cc4bib28] Choi A, Shin H (2017). Photoplethysmography sampling frequency: pilot assessment of how low can we go to analyze pulse rate variability with reliability?. Physiol. Meas..

[pmeaac6cc4bib60] Consumer Technology Association (2018). https://shop.cta.tech/collections/standards/products/physical-activity-monitoring-for-heart-rate.

[pmeaac6cc4bib37] Elgendi M (2014). Detection of c, d, and e waves in the acceleration photoplethysmogram. Comput. Methods Programs Biomed..

[pmeaac6cc4bib38] Elgendi M, Norton I, Brearley M, Abbott D, Schuurmans D (2014). Detection of a and b waves in the acceleration photoplethysmogram. Biomed. Eng. Online.

[pmeaac6cc4bib4] Esmaelpoor J, Moradi M H, Kadkhodamohammadi A (2021). Cuffless blood pressure estimation methods: physiological model parameters versus machine-learned features. Physiol. Meas..

[pmeaac6cc4bib14] Fallow B A, Tarumi T, Tanaka H (2013). Influence of skin type and wavelength on light wave reflectance. J. Clin. Monit. Comput..

[pmeaac6cc4bib19] Fine J, Branan KL, Rodriguez AJ, Boonya-Ananta T, Ajmal, Ramella-Roman JC, McShane MJ, Coté GL. (2021). Sources of inaccuracy in photoplethysmography for continuous cardiovascular monitoring. Biosensors.

[pmeaac6cc4bib21] Grabovskis A, Marcinkevics Z, Rubins U, Kviesis-Kipge E (2013). Effect of probe contact pressure on the photoplethysmographic assessment of conduit artery stiffness. J. Biomed. Opt..

[pmeaac6cc4bib31] Guo Z, Ding C, Hu X, Rudin C (2021). A supervised machine learning semantic segmentation approach for detecting artifacts in plethysmography signals from wearables. Physiol. Meas..

[pmeaac6cc4bib27] Hartmann V, Liu H, Chen F, Qiu Q, Hughes S, Zheng D (2019). Quantitative comparison of photoplethysmographic waveform characteristics: effect of measurement site. Front. Physiol..

[pmeaac6cc4bib9] Hultman M, Johansson I, Lindqvist F, Ahlström C (2021). Driver sleepiness detection with deep neural networks using electrophysiological data. Physiol. Meas..

[pmeaac6cc4bib50] Huthart S, Elgendi M, Zheng D, Stansby G, Allen J (2020). Advancing PPG signal quality and know-how through knowledge translation—from experts to student and researcher. Front. Digit. Health.

[pmeaac6cc4bib56] Johnson A E W, Pollard T J, Shen L, Lehman L H, Feng M, Ghassemi M, Moody B, Szolovits P, Anthony Celi L, Mark R G (2016). MIMIC-III, a freely accessible critical care database. Sci. Data.

[pmeaac6cc4bib46] Khalid S G, Liu H, Zia T, Zhang J, Chen F, Zheng D (2020). Cuffless blood pressure estimation using single channel photoplethysmography: a two-step method. IEEE Access.

[pmeaac6cc4bib16] Khan Y, Han D, Ting J, Ahmed M, Nagisetty R, Arias A C (2019). Organic multi-channel optoelectronic sensors for wearable health monitoring. IEEE Access.

[pmeaac6cc4bib55] Lee H C, Jung C W (2018). Vital recorder- a free research tool for automatic recording of high-resolution time-synchronised physiological data from multiple anaesthesia devices. Sci. Rep..

[pmeaac6cc4bib30] Lee J, Jang D H, Park S, Cho S H (2018). A low-power photoplethysmogram-based heart rate sensor using heartbeat locked loop. IEEE Trans. Biomed. Circuits Syst..

[pmeaac6cc4bib12] Lemay M, Bertschi M, Sola J, Renevey P, Genzoni E, Proença M, Ferrario D, Braun F, Parak J, Korhonen I. (2021). Applications of Optical Cardiovascular Monitoring. Wearable Sensors: Fundamentals, Implementation and Applications.

[pmeaac6cc4bib6] Li Q, Li Q, Cakmak A S, Poian G D, Bliwise D L, Vaccarino V, Shah A J, Clifford G D (2021). Transfer learning from ECG to PPG for improved sleep staging from wrist-worn wearables. Physiol. Meas..

[pmeaac6cc4bib34] Liang Y, Elgendi M, Chen Z, Ward R (2018). An optimal filter for short photoplethysmogram signals. Sci. Data.

[pmeaac6cc4bib43] Lin W H, Li X, Li Y, Li G, Chen F (2020). Investigating the physiological mechanisms of the photoplethysmogram features for blood pressure estimation. Physiol. Meas..

[pmeaac6cc4bib11] Liu H, Allen J, Khalid S G, Chen F, Zheng D (2021). Filtering-induced time shifts in photoplethysmography pulse features measured at different body sites: the importance of filter definition and standardization. Physiol. Meas..

[pmeaac6cc4bib13] Liu J, Yan B P, Zhang Y, Ding X, Su P, Zhao N (2019). Multi-wavelength photoplethysmography enabling continuous blood pressure measurement with compact wearable electronics. IEEE Trans. Biomed. Eng..

[pmeaac6cc4bib33] Marozas V, Charlton P H (2021).

[pmeaac6cc4bib17] Matsumura K, Toda S, Kato Y (2020). RGB and near-infrared light reflectance/transmittance photoplethysmography for measuring heart rate during motion. IEEE Access.

[pmeaac6cc4bib52] McCarthy C, Pradhan N, Redpath C, Adler A (2016). Validation of the empatica E4 wristband.

[pmeaac6cc4bib49] Mishra T (2020). Pre-symptomatic detection of COVID-19 from smartwatch data. Nat. Biomed. Eng..

[pmeaac6cc4bib42] Mohagheghian F (2022). Optimized signal quality assessment for photoplethysmogram signals using feature selection. IEEE Transactions on Biomedical Engineering.

[pmeaac6cc4bib58] Mühlen J M (2021). Recommendations for determining the validity of consumer wearable heart rate devices: expert statement and checklist of the INTERLIVE network. Br. J. Sports Med..

[pmeaac6cc4bib59] Mukkamala R, Yavarimanesh M, Natarajan K, Hahn J O, Kyriakoulis K G, Avolio A P, Stergiou G S (2021). Evaluation of the accuracy of cuffless blood pressure measurement devices: challenges and proposals. Hypertension.

[pmeaac6cc4bib40] Orphanidou C, Bonnici T, Charlton P, Clifton D, Vallance D, Tarassenko L (2015). Signal-quality indices for the electrocardiogram and photoplethysmogram: derivation and applications to wireless monitoring. IEEE J. Biomed Health Inform..

[pmeaac6cc4bib7] Ouyang V, Ma B, Pignatelli N, Sengupta S, Sengupta P, Mungulmare K, Fletcher R R (2021). The use of multi-site photoplethysmography (PPG) as a screening tool for coronary arterial disease and atherosclerosis. Physiol. Meas..

[pmeaac6cc4bib44] Peralta E, Lazaro J, Bailon R, Marozas V, Gil E (2019). Optimal fiducial points for pulse rate variability analysis from forehead and finger photoplethysmographic signals. Physiol. Meas..

[pmeaac6cc4bib35] Pilt K, Ferenets R, Meigas K, Lindberg L-G, Temitski K, Viigimaa M (2013). New photoplethysmographic signal analysis algorithm for arterial stiffness estimation. Sci. World J..

[pmeaac6cc4bib24] Prinable J B, Foster J M, McEwan A L, Young P M, Tovey E, Thamrin C (2017). Motivations and key features for a wearable device for continuous monitoring of breathing: a web-based survey. JMIR Biomed. Eng..

[pmeaac6cc4bib23] Radha M (2019). Estimating blood pressure trends and the nocturnal dip from photoplethysmography. Physiol. Meas..

[pmeaac6cc4bib48] Radin J M, Wineinger N E, Topol E J, Steinhubl S (2020). Harnessing wearable device data to improve state-level real-time surveillance of influenza-like illness in the USA: a population-based study. Lancet Digit. Health.

[pmeaac6cc4bib39] Rajala S, Lindholm H, Taipalus T (2018). Comparison of photoplethysmogram measured from wrist and finger and the effect of measurement location on pulse arrival time. Physiol. Meas..

[pmeaac6cc4bib54] Reiss A, Indlekofer I, Schmidt P, Van Laerhoven K (2019). Deep PPG: large-scale heart rate estimation with convolutional neural networks. Sensors.

[pmeaac6cc4bib22] Scardulla F, D’acquisto L, Colombarini R, Hu S, Pasta S, Bellavia D (2020). A study on the effect of contact pressure during physical activity on photoplethysmographic heart rate measurements. Sensors.

[pmeaac6cc4bib53] Schmidt P, Reiss A, Duerichen R, Van Laerhoven K (2018). Introducing WeSAD, a multimodal dataset for wearable stress and affect detection.

[pmeaac6cc4bib57] Stergiou G S (2018). A universal standard for the validation of blood pressure measuring devices: association for the Advancement of Medical Instrumentation/European Society of Hypertension/International Organization for Standardization (AAMI/ESH/ISO) Collaboration Statement. Hypertension.

[pmeaac6cc4bib51] Takazawa K, Tanaka N, Fujita M, Matsuoka O, Saiki T, Aikawa M, Tamura S, Ibukiyama C (1998). Assessment of vasoactive agents and vascular aging by the second derivative of photoplethysmogram waveform. Hypertension.

[pmeaac6cc4bib25] Wolling F, Van Laerhoven K (2018). Fewer samples for a longer life span.

[pmeaac6cc4bib3] Xing X, Ma Z, Xu S, Zhang M, Zhao W, Song M, Dong W-F (2021). Blood pressure assessment with in-ear photoplethysmography. Physiol. Meas..

[pmeaac6cc4bib45] Xing X, Ma Z, Zhang M, Gao X, Li Y, Song M, Dong W-F F (2020). Robust blood pressure estimation from finger photoplethysmography using age-dependent linear models. Physiol. Meas..

[pmeaac6cc4bib32] Zhang Z, Pi Z, Liu B (2015). TROIKA: a general framework for heart rate monitoring using wrist-type photoplethysmographic signals during intensive physical exercise. IEEE Trans. Biomed. Eng..

